# Description of a New Telonemia Genus and Species With Novel Observations Providing Insights Into Its Hidden Diversity

**DOI:** 10.1111/jeu.70050

**Published:** 2025-10-23

**Authors:** Helena Mostazo‐Zapata, Alex Gàlvez‐Morante, Cédric Berney, Xènia Maya‐Figuerola, Cristiana Sigona, David López‐Escardó, Elisabet L. Sà, Dolors Vaqué, Daniel J. Richter

**Affiliations:** ^1^ Institut de Biologia Evolutiva (CSIC‐Universitat Pompeu Fabra) Barcelona Spain; ^2^ Department of Marine Biology and Oceanography Institute of Marine Sciences (ICM), Barcelona Spain

**Keywords:** eukaryotic diversity, phylogeny, protists, taxonomy, Telonemia

## Abstract

Telonemia is a fascinating and understudied group of microbial eukaryotes known to have a vast diversity that is still uncharacterized. In fact, although their phylogenetic position is still actively debated, their diversity and biology are largely unexplored: to date, there are only seven described species in three genera, although there are estimated to be hundreds more unknown lineages. Here, we describe the isolation and characterization of two new strains, including a new genus (*Hyaliora molinica* n. gen. n. sp.) and a new species (*Telonema blandense* n. sp.), and the re‐isolation of a previously characterized telonemid, *Telonema subtile*, accompanied by new behavioral observations. We present morphological measurements highlighting differences among the isolates and a phylogenetic tree incorporating their 18S rRNA gene sequences. Furthermore, key aspects of their cell biology and structure are highlighted to provide insights into the evolution of their potential sister groups. Since they are relevant not only phylogenetically but also play a crucial role in food webs with some abundant representatives in aquatic ecosystems, the findings of this study provide further sampling and culturing of Telonemia to increase the knowledge of the hidden diversity and evolution of this mysterious group.

## 1 Introduction

Telonemia, commonly referred to as “telonemids,” is a poorly characterized eukaryotic group that was previously identified as the sister to the well‐established supergroup Sar (Stramenopiles, Alveolata, and Rhizaria) (Adl et al. 2019), which may comprise up to half of eukaryote species diversity (Grattepanche et al. 2018). Together, telonemids and Sar would form the TSAR group (Strassert et al. 2019). However, several recent phylogenomic studies have failed to recover the TSAR supergroup, placing Telonemia instead as the sister group to Haptista, despite having varying levels of support (Eglit et al. 2024; Torruella et al. 2025; Yazaki et al. 2022). Therefore, the phylogenetic position of this group remains uncertain. Sar and Haptista are both important eukaryotic groups, so in both cases, telonemids represent an opportunity to study the origin and evolution of their morphological synapomorphies and cellular innovations, as well as providing insights into eukaryotic diversification (Tikhonenkov et al. 2022).

Until now, there have been only seven characterized species of Telonemia in three different genera, although they were initially described over a century ago. The first species, *Telonema subtile* (also known as *T. subtilis* , a homotypic synonym), was described in 1913 by Griessmann from the marine habitat (more specifically, from crude cultures of *Ulva* and red algae from Roscoff, France, and Naples, Italy) as a relatively small (6–8 μm long), colorless, elliptical, rigid‐bodied flagellate without a contractile vacuole and with no close relationship with other known flagellates (Griessmann 1913). Another report on this species was published a few decades later, providing a further detailed morphological description with light microscopy, which incorrectly placed Telonemia within the now‐outdated family Cyathomonadidae (Cryptophyta, Chromista) due to its morphological similarity (Hollande and Cachon 1950), followed by several further studies (Vørs 1992, 1993). It was not until 2013 that a more detailed characterization was finally made, specifying its structural characteristics: cells contain mitochondria with tubular cristae, extrusomes, a telonemosome (a unique organelle considered to be a vesicle containing immature flagellar hairs), a nucleus, a multilayered cytoskeleton, and adhesive fibers (Yabuki et al. 2013). It took almost a century from the first description to isolate and characterize a second species, from the surface marine waters of the inner Oslo fjord, described as *T. antarcticum* (Klaveness et al. 2005), although in 2015 it was renamed as *Lateronema antarctica* due to its morphological and genetic differences with *Telonema*, resulting in a second genus (Cavalier‐Smith et al. 2015). Subsequently, in 2022, a new study was published describing three new species of Telonema (*T. rivulare*, *T. papanine*, and *T. tenere*) and two new species within the novel genus *Arpakorses: A. versatilis* and *A. idiomastiga* (Tikhonenkov et al. 2022). Very recently, *Microkorses curacao* was described, a new species belonging to a new genus (Zlatogursky et al. 2025). Altogether, these represent a very low percentage of telonemid diversity, as there are estimated to be over a hundred marine and freshwater undescribed lineages of telonemids (Bråte et al. 2010).

Currently, our knowledge of telonemids comes from previous studies that indicate that they are phagotrophic heterotrophic biflagellate protists of pyriform shape, with flagella emerging on opposite sides of a short protruding antapical rostrum or proboscis (Klaveness et al. 2005) (Shalchian‐Tabrizi et al. 2006). We note that a later study (Tikhonenkov et al. 2022) defined the flagellar pole as the “apical” end of the cell, but here the original description will be used. As described above, their unique structural characteristics are also a shared feature (Yabuki et al. 2013; Klaveness et al. 2005). This intricate cytoskeleton, among other structures and morphologies, might mean telonemids have retained ancestral conditions lost in other sister groups (Klaveness et al. 2005).

Due to their wide distribution and abundance, telonemids most likely play important ecological functions in both freshwater (Boukheloua et al. 2024) and marine ecosystems (Shalchian‐Tabrizi et al. 2006). They feed on a wide range of bacteria and pico‐ to nanophytoplankton (Bråte et al. 2010), and it seems they do not survive without their eukaryotic prey (Tikhonenkov et al. 2022). This feeding behavior positions them as important consumers within the microbial food web, contributing to nutrient cycling and energy flow in marine ecosystems.

In this study, three new strains of telonemids have been isolated. These include a proposed new genus and a new species from Mediterranean marine samples. In addition, *Telonema subtile* has been reisolated from Antarctic marine samples. A detailed description of their morphology and behaviors is presented together with statistically supported morphological differences between these three strains, accompanied by a phylogeny built with available 18S ribosomal RNA gene sequences of telonemids.

## 2 Materials and Methods

### 2.1 Isolation and Culture Maintenance

Cultures of *Hyaliora molinica* n. gen n. sp. strain BEAP0326 and *Telonema blandense* n. sp. strain BEAP0082 were isolated by diluting an initial sample from subsurface water sampled at the Blanes Bay Microbial Observatory (BBMO), a station located in the NW Mediterranean Sea about 1 km offshore (41°40′ N, 2°48′ E) over a water column of 20 m depth in February 2024. The isolates of *H. molinica* were kept at 17°C with 12 h light:dark cycles using RS medium in a ratio of 1 (Nutrient Media Component—P5) to 50 (Non‐Nutrient Media Component—P4) (Sigona et al., in preparation; https://mediadive.dsmz.de/medium/P4 and https://mediadive.dsmz.de/medium/P5) and contained a mix of unidentified prokaryotes and other eukaryotes (including 18S rRNA sequences from Bicosoecida and MAST representatives, although 18S sequencing was not exhaustive). *Hyaliora* sp. (BEAP0010) was also isolated by dilution from the same sampling point in Blanes Bay, but the culture died before any morphological characterization could be performed. The cultures of *T. blandense* were stored at 17°C in darkness using RS medium 1:10 and included a mix of unidentified prokaryotes and other eukaryotes (including 18S rRNA sequences from MAST representatives, although 18S sequencing was not exhaustive). *Telonema subtile* strain BEAP0314 was isolated from Antarctic surface waters (66°20′S, 67°30′W) sampled on February 22, 2023, over a water column of 5 m depth and was kept at 4°C in darkness using RS medium 1:100.

### 2.2 18S rRNA Gene Sequencing

A sample of 50 mL per culture was used for 18S cloning. The cells were resuspended in the pellet after being centrifuged for 20 min at 13000 × g. DNA was extracted using the DNeasy PowerSoil Pro kit (QIAGEN). The 18S rRNA genes were amplified by PCR using universal eukaryotic primers 42F‐1747R (5′ CTCAARGAYTAAGCCATGCA 3′ ‐ 5′ CCTTCYGCAGGTTCACCTAC 3′) for *T. blandense* (BEAP0082), *H. molinica* (BEAP0326), and *Hyaliora* sp. (BEAP0010) and 82F‐1732R (5′ GAAACTGCGAATGGCTC 3′ ‐ 5′ ACCTACGGAAACCTTGTTACG 3′) for *T. subtile* (BEAP0314). Amplification products were purified using the NZYGelpure kit (NZYTech), cloned using the TOPO‐TA Cloning kit (Invitrogen), and transformed into *E. coli* cells following LacZα‐complementation. Positive clones were selected and amplified by PCR with vector‐specific primers M13F‐M13R (5′ GTAAAACGACGGCCAGT 3′ ‐ 5′ CAGGAAACAGCTATGAC 3′). Sequencing was performed by Eurofins Genomics using Sanger sequencing. The resulting sequences were base called using phred (Ewing et al. 1998) with the parameters “‐trim_alt ‘’ ‐trim_cutoff 0.01” and assembled with phrap (De la Bastide and McCombie 2007 *)* with the parameter “‐repeat_stringency 0.4” and the consensus sequence was exported with consed (Gordon et al. 1998). The assembled 18S rRNA gene sequences of our isolates (BEAP0010, BEAP0082, BEAP0314: each a consensus of 9 sequenced clones; BEAP0326: consensus of 5 sequenced clones) have been deposited in GenBank with the accession codes PV654195‐PV654198. These sequences were compared with EukRibo version 1.0 (Berney et al. 2022) through BLAST (Camacho et al. 2009) so as to confirm that they belong to the observed Telonemia isolates and not any other eukaryote in the cultures.

### 2.3 Sequence Alignments and Phylogenetic Analyses

To place the new isolates in the phylogenetic tree of Telonemia, we built an 18S rRNA alignment consisting of the four new sequences we obtained, all available telonemid 18S rRNA sequences from described species, and environmental clones representative of the undescribed telonemid diversity. We recovered these environmental clones both from GenBank (Sayers et al. 2025) and from a long‐read environmental survey of various habitat types published by Jamy et al. (2022). Members of two independent eukaryotic groups close to Telonemia (Haptophyta and Provora) were used as outgroups. For taxa with transcriptome or genome data available in EukProt (Richter et al. 2022), a complete 18S rRNA sequence was extracted from the assembled transcriptome contigs or genome shotgun sequences and was used to replace the corresponding GenBank sequence to ensure the best possible 18S rRNA coverage and sequence quality (Table S1). A second alignment of a larger portion of the ribosomal operon (matching the fragment amplified in the Jamy et al. (2022) dataset, which goes from before the V4 region of the 18S rRNA to about two‐thirds into the 28S rRNA) was built for members of the TEL‐1 clade within Telonemia. It includes sequences from all the described members of the TEL‐1 clade for which all or part of the ribosomal operon could be extracted from assembled transcriptome contigs, as well as the environmental clones from the Jamy et al. (2022) dataset already present in the 18S rRNA alignment.

Both alignments were generated manually in BioEdit version 7.2.5 (Hall 1999). Ambiguously aligned positions were manually trimmed following secondary structure models for the 18S rRNA (Wuyts et al. 2000) and 5.8S + 28S rRNA (Ben Ali et al. 2001). More positions had to be excluded in the first alignment (18S rRNA only) because it contains a wider diversity of organisms with more distantly related sequences. By contrast, organisms within the TEL‐1 clade have conserved sequences that remain alignable even in the most variable regions of both the 18S and 28S rRNA, so that very few positions had to be trimmed. Next, a maximum‐likelihood phylogenetic tree was inferred from each dataset with RAxML version 8.2.10 (Stamatakis 2014) using the GTRGAMMA model, and statistical support at internal nodes was estimated with the rapid bootstrapping algorithm with 1000 pseudoreplicates. Finally, the trees were visualized and edited with iTOL (Letunic and Bork 2021).

### 2.4 Morphological Analysis

Still and time‐lapse images were collected using differential interference contrast (DIC) with a Zeiss Axiovert Inverted microscope equipped with a 63× oil‐immersion lens. Subsequently, digital images were processed using Fiji software (Schneider et al. 2012) (Schindelin et al. 2012).

Measurements of cell length, cell width, food vacuole diameter, and flagella length were taken in 20 cells of each strain and analyzed with R Studio (RStudio Team 2020). For flagellar length classification, the longer visible flagellum was designated as the “long flagellum,” while the shorter was identified as the “short flagellum.” Packages used were tidyverse (Wickham et al. 2019), ggplot2 (Wickham 2016), and patchwork (Pedersen 2024) for data analysis and the generation of graphs. To identify significance in the difference between the three isolates, ANOVA tests were run with a 0.05 significance level. Normality of residuals was checked using Shapiro–Wilk and homogeneity of variances with the Bartlett test. In the cases in which ANOVA was significant, multiple comparisons were performed with the Tukey test to find interspecific differences.

For scanning electron microscopy (SEM), cells were fixed using 2.4 mL of a 3% glutaraldehyde solution, diluted in culture medium from a 25% stock. Cells were incubated for 3 h at room temperature and then seeded on membranes with pores sized 0.8 μm (WHA10417301, MERCK Chemicals and Life Science) via filtering. The samples were dehydrated in a graded ethanol series and critical‐point dried with liquid carbon dioxide in a Leica EM CPD300 unit (Leica Microsystems, Austria). The dried filters were mounted on stubs with colloidal silver and then were sputter‐coated with gold in a Q150R S (Quorum Technologies Ltd.) and observed either with a Hitachi S3500N scanning electron microscope (Hitachi High Technologies Co. Ltd., Japan) at an accelerating voltage of 5 kV or with a Hitachi SU8600 field emission scanning electron microscope (Hitachi High Technologies Co. Ltd., Japan) in the Electron Microscopy Service of the Institute of Marine Science (ICM‐CSIC), Barcelona.

## 3 Results

### 3.1 Phylogenetic Relationships Within Telonemia

**FIGURE 1:**
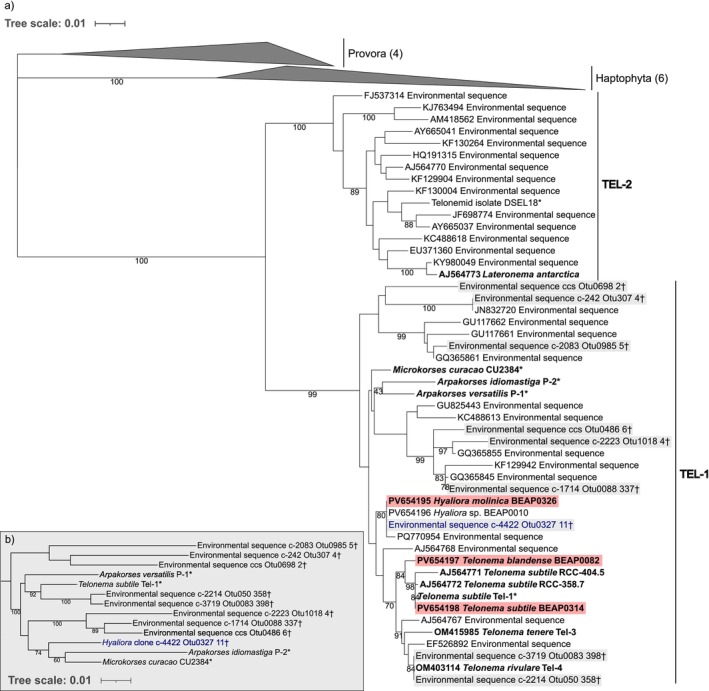
Maximum‐likelihood phylogenetic trees showing the position of our new isolates within Telonemia based on complete 18S rRNA sequences (a) and highlighting the position of *Hyaliora* compared to other described taxa within the TEL‐1 clade based on a portion of the whole ribosomal operon (b). Both trees were inferred with RAxML. Numbers at the nodes represent automatic thorough bootstrap support after 1000 pseudoreplicates. Only values above 70 are shown, plus the value for the sister relationship of the two *Arpakorses* species in the 18S only tree. (a) Telonemia phylogeny based on 1592 unambiguously aligned positions of the 18S rRNA, with members of Haptophyta and Provora used as outgroups. Names in bold represent formally described species, and taxa in red are the species examined in detail in this work. Gray boxes highlight taxa and environmental clones present in both panels. (b) Phylogeny of the TEL‐1 clade based on 1224 (18S), 154 (5.8S), and 2327 (28S) unambiguously aligned positions of the ribosomal operon. The tree is rooted consistently with the 18S phylogeny from panel a. Sequences of described species extracted from transcriptome assemblies are marked with *, while selected clones from the long‐read environmental survey of Jamy et al. (2022) are marked with † (see Methods for details). The sequence labeled in blue is the same environmental clone in both trees and is used as a representative of the new genus *Hyaliora* in panel (b).

We reconstructed a phylogenetic tree containing the 18S rRNA gene sequences of our three new isolates of telonemids, together with all currently available telonemid 18S sequences from described species and environmental clones representative of undescribed telonemid diversity (Figure 1a). Based on its 18S rRNA gene sequence, one of the three isolates (BEAP0314) corresponds to *T. subtile* , as it is 100% identical to *Telonema subtile* Tel‐1 (Tikhonenkov et al. 2022). Two other strains from the Roscoff Culture Collection probably also belong to *T. subtile* ; the observed differences in their sequences may be errors. A second isolate (BEAP0082) also branches inside the genus *Telonema* but separately from previously described species; it is identified as a new species *Telonema blandense* n. sp. Lastly, a new genus *Hyaliora molinica* n. gen n. sp. is proposed for the third isolate (BEAP0326), as our 18S phylogeny suggests that it represents a separate lineage that does not branch with any other described genus of telonemids. It clusters with three 18S rRNA sequences: one from a fourth isolate (BEAP0010) that died before we could study it morphologically, one GenBank environmental clone (PQ770954) from a river estuary in China, and one long‐read environmental clone from the dataset generated by Jamy et al. (2022) from a North Sea euphotic marine water surface sample at 5 m depth. Because resolving the internal relationships within Telonemia is difficult with 18S rRNA sequences alone (as evidenced by the low bootstrap support at many internal nodes in Figure 1a), we then constructed a second dataset including a larger part of the ribosomal operon matching the complete fragment amplified in the Jamy et al. (2022) study. For this phylogenetic analysis, we focused on the TEL‐1 clade in order to increase the number of unambiguously aligned positions that could be used to infer the tree (Figure 1b). This analysis confirms that the proposed new genus *Hyaliora* (represented by the environmental clone c‐4422_Otu0327_11 from the Jamy et al. (2022) dataset) branches separately from described species and cannot be assigned to any described genus. However, it also shows the two *Arpakorses* species branching separately, the type species *A. versatilis* being weakly placed as sister to the genus *Telonema*, while *A*. *idiomastiga* is weakly placed as a sister to the genus *Microkorses*.

### 3.2 Cell Size and Flagellar Lengths: Intraspecific Variation and Interspecific Differences

**FIGURE 2:**
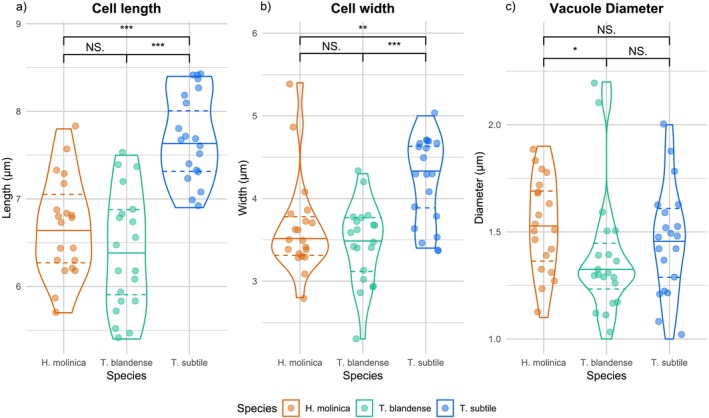
Distribution of cell length (a), cell width (b), and vacuole diameter (c) between the three isolates of this study. Data were collected from 20 cells using differential interference contrast (DIC) microscopy and measured using Fiji Software. *p < 0.05; **p < 0.01; ***p < 0.001; N.S: Nonsignificant (*p* ≥ 0.05).

In terms of cell size, *T. subtile* is significantly larger than *T. blandense* and *H. molinica* (length: *p* = 10^−9^; width: *p* = 10^−5^), which are not significantly different from one another (Figure 2a,b). As expected, food vacuole diameters are not significantly different among the three strains (*p* = 0.3, Figure 2c), as they are likely to depend on food availability and prey size (Tikhonenkov et al. 2022).

**FIGURE 3:**
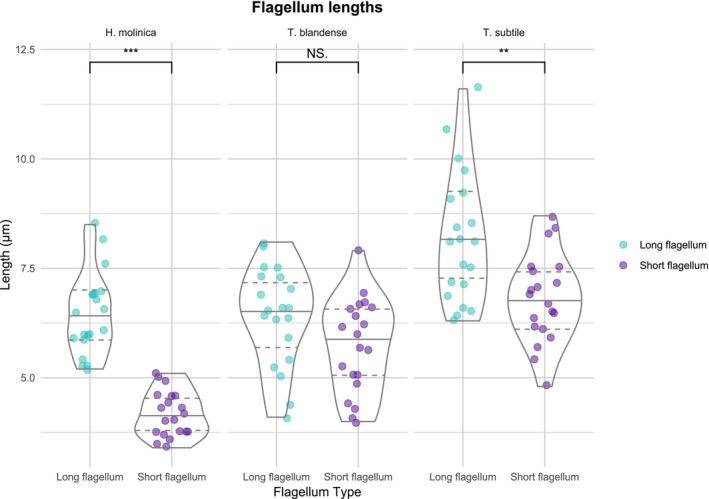
Distribution of flagella length between the three isolates of this study. Mean values are indicated by a blue point. Data were collected from 20 cells with differential interference contrast (DIC) microscopy and measured using Fiji Software. **p < 0.01; **p < 0.001; N.S.: Nonsignificant (*p* ≥ 0.05).

Figure 3 presents the distribution of values for the lengths of both the long and short flagella in each species (with the longer of the two flagella considered the “long flagellum”; see Methods). Multiple comparisons reveal *T. subtile* has a larger mean flagella length, followed by *T. blandense* and *H. molinica*. Moreover, it is clear that both *H. molinica* and *T. subtile* possess significantly uneven flagella (*p* = 0.009). However, in *T. blandense*, both flagella would be equal according to this data.

### 3.3 External Morphology, Behavior, and Ecology of the Isolates

We observed that all three telonemid strains in this study did not survive without another eukaryote present in the culture, presumably as prey. This has also been previously reported by Tikhonenkov et al. (2022) in other isolates within Telonemia.

#### 3.3.1 *Hyaliora molinica* n. gen. n. sp.

**FIGURE 4:**
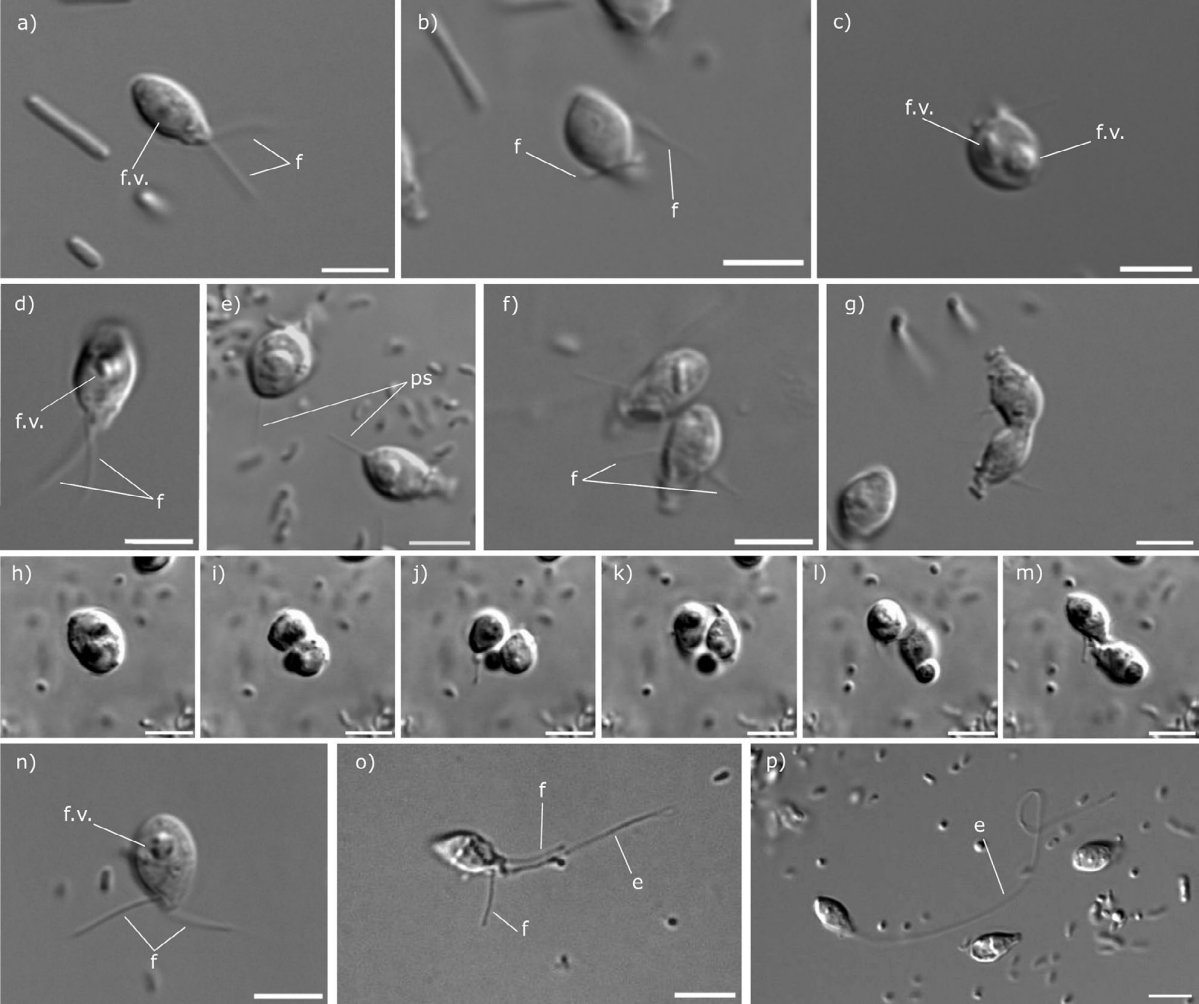
External morphology of the three telonemids isolated in this study with differential interference contrast (DIC) microscopy. *Hyaliora molinica* n. gen n. sp., free cell (a), attached to the surface (b) and in the first stages of division (c). *Telonema blandense* n. sp., free cell (d), two cells with pseudopodia (e), two cells attached to the surface (f), and in the last stages of division (g) and sequence of possible vacuole reabsorption (h–m). *Telonema subtile*, free cell (n) and with an elongating structure (o–p). Abbreviations: E: elongating structure; f: flagellum; f.v.: vacuole. Scale bar 5 μm.

**FIGURE 5:**
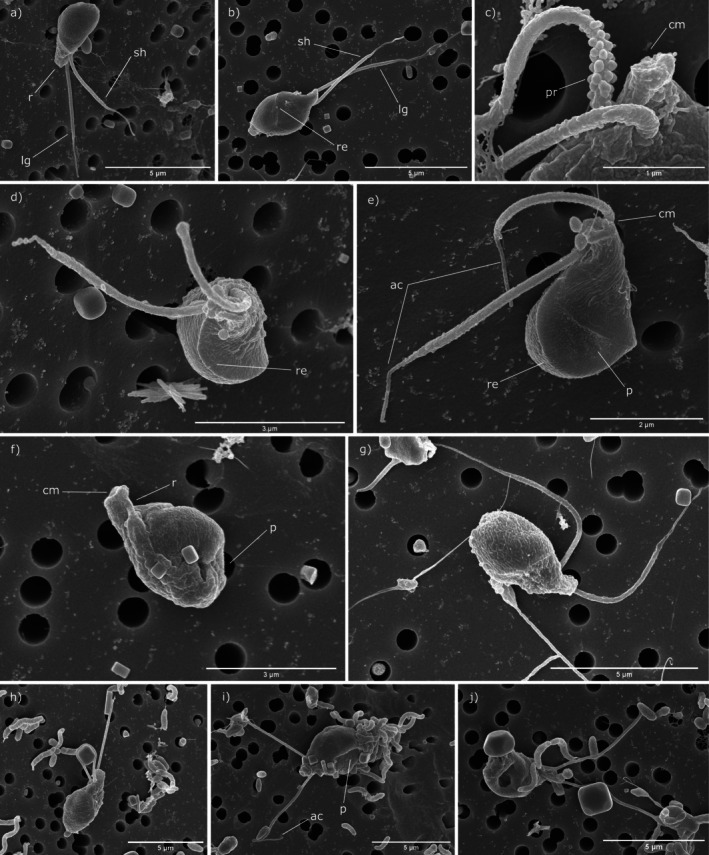
External morphology of the three telonemids isolated in this study with scanning electron microscopy (SEM). *Hyaliora molinica* n. gen. n. sp. (a‐e), *Telonema blandense* n. sp. (f‐g), and *Telonema subtile* (h‐j). Abbreviations: Ac: acroneme; cm: cytostome; lg: long flagellum; p: pit; pr: protuberance; r: rostrum; re: relief; sh: short flagellum.

The cell body of *H. molinica* (Figures 4a–c and 5a–e) is drop‐shaped with a rostrum containing a cytostome in the antapical part of the cell (Figure 5c,e), with a cell length of 5.7–7.8 μm and 2.8–5.4 μm in cell width. Two acronematic flagella emerge from the antapical part, with one of them (3.4–5.1 μm) significantly shorter than the other (5.3–8.5 μm) (Figures 3 and 4a). Mastigonemes were either missing or not detected in our images. In seven of 13 cells analyzed by scanning electron microscopy, we observed numerous protuberances in the base of the flagella (Figure 5c), although this observation may be an artifact of sample preparation. On the cell surface, a relief can be seen in all cells (Figure 5b,d,e) as well as a pit on one side of the cell (Figure 5e); the latter is a distinctive trait of telonemids (Tikhonenkov et al. 2022), (Shalchian‐Tabrizi et al. 2006). A food vacuole is located between the center and the posterior part of the cell (Figure 4a,c) and, in well‐fed cells, it can be quite prominent, as reported by Klaveness et al. (2005) and Tikhonenkov et al. (2022) in other species of Telonemia. In those well‐fed cells, the shape is rounder instead of pyriform. Cells swim, twirling around their longitudinal axis with their flagella directed away from the direction of movement, although when sedentary, the flagella are wrapped around the cell body, with both in the same direction (Figure 4b). This behavior has been observed in species of the genus *Telonema*, but not in other genera (Tikhonenkov et al. 2022). Cell division is longitudinal, as described in all other characterized species of telonemids (Tikhonenkov et al. 2022).

#### 3.3.2 *Telonema blandense* n. sp.

*Telonema blandense* n. sp. is a proposed new species in the previously described genus *Telonema* (Figures 4d–m and 5f,g) that possesses cell length (5.4–7.4 μm) and width dimensions (2.3–4.3 μm) similar to those of *H. molinica*. It has two emerging independent acronematic flagella that are not significantly different in length (3.9–7.9; 4.1–8.1 μm) (Figures 4d and 5g). No mastigonemes were detected. A cytostome in the rostrum can also be found in this species (Figure 5f) in addition to the pit in the surface. The food vacuole is consistently slightly closer to the posterior part of the cell in comparison to *H. molinica* (Figure 4d). A 0.7–5.6 μm long posterior pseudopodium was observed in sessile cells, where it appeared to provide substrate attachment (Figure 4e, Video S1). They also swim spinning in their longitudinal axis with flagella backwards and wrap the flagella when not moving (Figure 4f). Cell division has been observed to be longitudinal (Figure 4g). Interestingly, in cells with a prominent vacuole, we observed a new and unusual behavior: they seem to eject their vacuole to later reabsorb it, possibly as a way to free up space inside the cell during division (Figure 4h–m). These structures have also been seen being ejected from the cell and floating freely in the medium (Video S2). To our knowledge, these observations have not previously been reported in any species of Telonemia.

#### 3.3.3 Telonema subtile

The body morphology and shape of *Telonema subtile* (Figures 4n–p and 5h–j) are similar to all other characterized species within the genus, but with significantly bigger dimensions compared to *T. blandense* (and to *H. molinica*), with a cell length of 7.0–8.4 μm and width of 3.4–5.0 μm. It has two uneven acronematic flagella, also significantly larger than the other species in this study (5.7–8.7 and 6.3–11.6 μm). The food vacuole is consistently located in the posterior part of the cell, as in *T. blandense* (Figure 4n). In this species, we have not observed wrapping of flagella when sedentary, consistent with previous studies (Tikhonenkov et al. 2022). However, a novel elongating structure in the antapical part independent from the flagella has been detected (Figure 4o,p, Video S3). The structure was observed to be up to 71.2 μm in length, or 59.6 μm longer than the longer of the two flagella. Its function remains unknown (see Discussion).

## 4 Discussion

The phylogenetic relevance of Telonemia has been previously highlighted (Strassert et al. 2019), despite the relative lack of information about its biology and diversity. In this study, we contributed to a better understanding of telonemids through the isolation and detailed analysis of a new genus, a new species of a previously characterized genus, and the novel observations made in an already known species. These new isolates add to the seven previously described species in the group.

Our phylogenetic analyses (Figure 1) demonstrate that all our new isolates belong to the TEL‐1 clade within Telonemia (as do all previously described species apart from *Lateronema antarctica*). Relationships within this clade are difficult to resolve with 18S rRNA sequences alone, and most internal nodes received low bootstrap support in our analysis (Figure 1a). To try to improve resolution within the TEL‐1 clade, we built a second dataset including a larger part of the ribosomal operon (matching the complete fragment amplified in the Jamy et al. (2022) study). This led to stronger support for the monophyly of the genus *Telonema* and strengthened the evidence that the lineage containing our isolate BEAP0316 should be described as a new genus (Figure 1b). However, it also led to a separation of the two described *Arpakorses* species. We note that this separation was also recovered by the phylogenomic analyses performed by Zlatogursky et al. 2025 (their Figure S1), with the type species *A. versatilis* branching closer to *T. subtile* than to *A*. *idiomastiga*. Given the morphological differences between the two *Arpakorses* spp., it is possible that the genus may not be monophyletic and may eventually need to be split into two separate genera. Evidently, further multigene data are needed to better elucidate phylogenetic relationships within telonemids, especially in the TEL‐1 clade.

The three new strains we present exhibit morphological similarities to previously described telonemids (Table 1), and they all possess the distinctive traits of the group. Nonetheless, there are some differences among them, such as a larger cell body size in *Telonema subtile* (Figure 2a). There are a few clear morphological features that distinguish *Hyaliora* from the recently proposed new genus *Microkorses* (Zlatogursky et al. 2025). First, there is a notable size difference: *Microkorses curacao* stands out for its small size (5–6 × 2.5–3 μm), which is smaller than all previously described telonemids, whereas *Hyaliora molinica* has size measurements (5.7–7.8 × 2.8–5.4 μm) more similar to other previously described species (Table 1). Similar to all *Telonema* spp. (with the exception of our isolate, *T. subtile* BEAP0314) and *A. idiomastiga*, *Microkorses* has flagella of equal length. The flagella of Microkorses also notably exceed its cell length. *Lateronema antarctica* and *A. versatilis* have flagella of unequal length, which exceed the length of the cell. In contrast to all of the above cases, Hyaliora has significantly uneven flagella that do not exceed their cell length. These characters, however, might not be discriminative enough, as different *T. subtile* isolates can differ in both respects (Table 1).

**TABLE 1:** Morphological comparison between described telonemids.

Species and isolate	Isolation site and habitat	Cell length (μm)	Cell width (μm)	Vacuole diameter (μm)	Flagella length (μm)	Uneven flagella	Flagella wrap around the cell	Other features
*Hyaliora molinica* BEAP0326 (This study)	Blanes, Spain (41°40′ N, 2°48′ E), marine	5.7–7.8	2.8–5.4	1.1–1.9	3.4–5.1; 5.3–8.5	Yes	Yes	Cell surface relief
*Telonema blandense* BEAP0082 (This study)	Blanes, Spain (41°40′ N, 2°48′ E), marine	5.4–7.4	2.3–4.3	1.0–2.2	3.9–7.9; 4.1–8.1	No	Yes	Presence of posterior pseudopodium and vacuole reabsorption
*Telonema subtile* BEAP0314 (This study)	Antarctica (69°19’S, 66°95’W), marine	7.0–8.4	3.4–5.0	1.0–1.9	5.7–8.7; 6.3–11.6	Yes	No	Elongating structure
*Telonema subtile* Tel‐1 (Tikhonenkov et al. 2022)	Kara Sea (75.888 N, 89.508 E), marine	7.0–9.4	3.5–6.1	NA	7.5–9.2	No	No	Cells swim with sharp jerks and alternately change motion from rectilinear to rotation
*Telonema papanine* Tel‐2 (Tikhonenkov et al. 2022)	Franz Josef Land archipelago, Russia (80°37′46.800 N, 56°53′45.500 E), freshwater	8.5–13.7	4.2–8.3	NA	7.4–10.9	No	Yes	Presence of pseudopodium to attach to substrate
*Telonema tenere* Tel‐3 (Tikhonenkov et al. 2022)	White Sea (66°29′58.78700 N, 35°9′42.20600 E), marine	5.8–10.2	3.5–7.2	NA	3.5–8.5	No	No	Cell body more elongated and slightly curved inwards; cannibalism observed
*Telonema rivulare* Tel‐4 (Tikhonenkov et al. 2022)	Sakhray River, Russia (44°10′26.700 N, 40°17′58.800 E), freshwater	10–14	5.0–9.5	NA	4.0–8.5	No	Yes	Relatively short flagella
*Arpakorses versatilis* P‐1 (Tikhonenkov et al. 2022)	Kara Sea (75.888 N, 89.508 E), marine	5.6–8.7	3.5–6.1	NA	7.7–15.5; 7.6–11.3	Yes	No	Cells attach to the substrate with one flagellum
*Arpakorses idiomastiga* P‐2 (Tikhonenkov et al. 2022)	Kara Sea (75.888 N, 89.508 E), marine	5.6–8.7	3.5–6.1	NA	3.3–7.2	No	No	Form cell clusters, free flagellum creates a flow of water
*Microkorses curacao* CU2384 (Zlatogursky et al. 2025)	Caribbean Sea (12.109 N, −68.953 E), marine	5–6	2.5–3	NA	Notably exceeding the cell length	No	NA	Tendency to attach to the substratum
*Lateronema antarctica* (Klaveness et al. 2005)	Inner Oslo fjord, Norway (59°25′00″ N 10°34′00″ E), marine	8–16	6–12	NA	Significantly longer than the cell	Yes	NA	Presence of alveoli in the surface

As the first species, *Telonema subtile*, was described over a century ago (Griessmann 1913) without an associated 18S rRNA sequence, it is impossible to determine with certainty whether previously identified isolates assigned to this species or genus belong to the genetic lineage that is now known as *T. subtile* or even to the genus *Telonema*. Consequently, all past morphological, ultrastructural, and behavioral observations not linked to an 18S sequence provide possible phenotypes within Telonemia; however, they cannot be certainly linked to a specific taxon, which may explain the observed morphological variations found among them. The isolate identified in this study as *T. subtile* and the isolate of Tikhonenkov et al. (2022) (Table 1) share the same sequence and exhibit sufficient morphological similarity to the original description; therefore, they are now classified as *Telonema subtile*, while the original organism described would have represented a more general characterization of Telonemia. Consistent with this idea, we note that the original description of this species by Griessmann indicated that the flagella were equal in length. The discrepancy with the observation of unequal flagellar lengths in our culture could therefore be explained by the fact that a subset of the cells does indeed have flagella of equal length, and by the possibility that Griessmann's description may have been based on a sample of cells that did not represent the variation present in the species.

Ultrastructurally, all studied species in this work show the morphology that defines this general description of Telonemia: biflagellated tear‐shaped unicellular organisms with a pit on the surface (Shalchian‐Tabrizi et al. 2006). Nevertheless, some exclusive traits have been observed in these new isolates, which might contribute to the understanding of the evolution of telonemids and their potential eukaryotic sister groups. For instance, in *Hyaliora molinica*, there is a clear relief on the surface of all cells. A notable feature in Telonemia is the presence of a very complex cytoskeleton organization in diverse layers. According to Cavalier‐Smith et al. (2015), two major parts of the row of anterior microtubules in *T. subtile* resemble the two posterior centriolar roots in Excavata, which might indicate that this highly intricate cytoskeleton is an ancestral condition kept by Telonemia and reduced differentially in the rest of eukaryotes (Strassert et al. 2019). It can be hypothesized that this relief on the surface is actually the belt or “corset,” the point where the anterior and posterior layers of the cellular peripheric cytoskeleton join. This belt is made of electronically dense material and tubulin microtubules (Tikhonenkov et al. 2022). On the other hand, there is the supposition that this relief is provoked by the so‐called “alveoli,” which are present in the *Lateronema* genus (Klaveness et al. 2005) and are a distinctive trait in the closely related group Alveolata (Cavalier‐Smith 2013), meaning that they could be homologous (Cavalier‐Smith 2003). These “alveoli” are flattened vesicles under the cortical membrane containing electron‐dense material (its composition varies depending on the group, but in *L. antarcticum* it was observed that they are crystalline structures (Klaveness et al. 2005)), and externally, their relief can be observed, which resembles the relief in *H. molinica*. Their function would be to reinforce the cellular cortex (Cavalier‐Smith et al. 2015). According to Takahashi et al. (2015), the common eukaryotic ancestor of the TSAR supergroup, Glaucophyta and Haptista, would have these structures present in its cell body.

Focusing on the elongating structure observed in *Telonema subtile*, previous studies have noted the presence of a pseudopodium in *Telonema papanine*, a recently characterized species from the same genus, which emerges from the posterior part and serves to anchor the cell to the substrate in sedentary cells (Tikhonenkov et al. 2022). We observed a morphologically similar pseudopodium in *T. blandense*, suggesting that it may serve the same function. However, there are morphological differences between the pseudopodia of *T. papanine* and *T. blandense* in comparison to our observations in *T. subtile* . In contrast to *T. papanine* and *T. blandense*, the elongating structure is longer, slightly thicker, and it undulates actively (Video S3). This suggests that, although the elongating structure of *T. subtile* might be involved in surface attachment, it could be somehow involved in environmental or prey sensing. Gliding locomotion is unlikely to be one of the functions, as cells have been seen only locally twisting rather than actively moving. Another possibility would be that it is a homologous structure to the haptonema present in haptophytes, which consists of a microtubule‐supported appendage that lies between two approximately equal flagella. Its functions, despite not being fully characterized, appear to be related to phagotrophic activity, surface attachment, and cell movement. Like the structure seen in *T. subtile* , it can elongate and shorten (Kawachi et al. 1991). Such structures were not characterized in previous descriptions of telonemids (Shalchian‐Tabrizi et al. 2006; Tikhonenkov et al. 2022; Yabuki et al. 2013). Further characterization of this structure is needed to fully understand its composition, origin, and function.

Regarding the vacuole reabsorption we observed in *Telonema blandense*, there are no previous reports on a similar process in any other described telonemid species. It may be an unusual behavior in well‐fed cells with a prominent vacuole that are in the process of division, as a way of freeing up space inside the cytoplasm. Another hypothesis would be that this event consists of extrusive organelles being discharged from the cell, since these structures have been reported to be present in ultrastructure analyses of *T. subtile* (Yabuki et al. 2013) and the *Arpakorses* genus (Tikhonenkov et al. 2022).

From the ecological point of view, there is evidence of a wide distribution of telonemids in marine ecosystems at high abundance (Klaveness et al. 2005). Previous studies describe telonemids as phagotrophic organisms that prey on a wide range of bacteria and pico‐ to nanophytoplankton (Bråte et al. 2010), which means they likely play a crucial role in ecosystems as a consumer of other microorganisms of similar or smaller size. Since none of the three species characterized in this study survived without other smaller eukaryotes present in the cultures, telonemids might be dependent on these prey–predator dynamics. Identical observations have been previously made in the genus *Arpakorses* (Tikhonenkov et al. 2022).

In conclusion, in this study, we shed an additional ray of light on the hidden diversity of Telonemia, which remains a mysterious phylum that may have relevant evolutionary importance in the history of the eukaryotic cell. Broader environmental sampling in both marine and freshwater ecosystems around the world is needed to establish more clonal cultures of uncharacterized new lineages and to continue to obtain new information on the already described telonemids. Also, further genomic and transcriptomic sequencing from clonal cultures and environmental samples is required to build a more solid and detailed phylogeny in order to fully comprehend the vast biodiversity in this enigmatic phylum.

## 5 Taxonomic Summary

Eukaryota, Diaphoretickes, Telonemia.

### 5.1 *Hyaliora* Mostazo‐Zapata, Gàlvez‐Morante, and Richter, n. gen.

Description: Biflagellated tear‐shaped protists with a rostral outgrowth and cytostome in the antapical part of the cell. They possess two uneven acronematic flagella. They are phagotrophic and have been observed not to survive without other eukaryotes.

Etymology: from Ancient Greek ὕαλος (“glass” or “transparent”) and derived from Latin ‐ora (“beauty” or “appearance”). Feminine.

Type species: *Hyaliora molinica*.

ZooBank registration: Described under the Zoological Code; ZooBank registration urn:lsid:zoobank.org:act:4E11A034‐447C‐4A94‐B94D‐B5BEC2A2CFCD.

### 5.2 *Hyaliora molinica* Mostazo‐Zapata, Gàlvez‐Morante, and Richter, n. sp.

Description: Cell average dimensions are 6.7 × 3.6 μm with significantly uneven acronematic flagella (3.4–5.1; 5.3–8.5 μm). The most common cell shape is tear‐shaped, although it varies depending on feeding conditions. They swim rotating around their longitudinal axis with their flagella directed backwards, although in sedentary cells, flagella are seen wrapping the cell body. Cell division is longitudinal.

Etymology: Species name refers to the first author's birth town (Molins de Rei).

Type locality: Subsurface water, Mediterranean Sea, Blanes Bay, Spain, 41°40′ N/2°40′ E.

Type material: The name‐bearing type (a hapantotype) is an SEM stub deposited in the Marine Biological Reference Collections (CBMR) at the Institut de Ciències del Mar (ICM‐CSIC, Barcelona, Spain) under the catalog/accession number ICMCBMR000699 (Guerrero et al. 2023). This material also contains other eukaryotes and uncharacterized prokaryotic species, which do not form part of the hapantotype.

Gene sequence: The 18S rRNA gene sequence of isolate BEAP0326 is deposited in GenBank as PV654195.

Cell culture: A culture containing *H. molinica* and other eukaryotes and bacterial species is publicly available and has been deposited in the Culture Collection of Algae and Protozoa (CCAP) with accession number CCAP 2621/1.

ZooBank registration: Described under the Zoological Code; ZooBank registration urn:lsid:zoobank.org:act:745F3D6A‐49D7‐425E‐A9EE‐5D0469245671.

### 5.3 *Telonema blandense* Mostazo‐Zapata, Gàlvez‐Morante, and Richter, n. sp.

Description: Pyriform or ovoid biflagellated protist of 6.4 × 3.4 μm with equal acronematic flagella (4.0–8.0; 4.1–8.1 μm). Cells wrap their flagella around the body when not moving and swim with flagella directed backwards. A long posterior pseudopodium can be found in sessile cells. Ejection of the food vacuole has been observed when longitudinally dividing.

Etymology: Species name refers to the sampling location “Blanes” (Lat.).

Type locality: Subsurface water, Mediterranean Sea, Blanes Bay, Spain, 41°40′ N/2°40′ E.

Type material: The name‐bearing type (a hapantotype) is an SEM stub deposited in the Marine Biological Reference Collections (CBMR) at the Institut de Ciències del Mar (ICM‐CSIC, Barcelona, Spain) under the catalog/accession number ICMCBMR000700 (Guerrero et al. 2023). This material also contains other eukaryotes and uncharacterized prokaryotic species, which do not form part of the hapantotype.

Gene sequence: The 18S rRNA gene sequence of isolate BEAP0082 is deposited in GenBank as PV654197.

Cell culture: A culture containing *T. blandense* and other eukaryotic and bacterial species is publicly available and has been deposited in the Culture Collection of Algae and Protozoa (CCAP) with accession number CCAP 2134/2.

ZooBank registration: Described under the Zoological Code; ZooBank registration urn:lsid:zoobank.org:act:169E250A‐DF24‐48FF‐A84D‐20EA72C2FD81.

This publication (work) has been registered with ZooBank as: urn:lsid:zoobank.org:pub:3FA06EE3‐60A1‐47D6‐9F00‐B8CED5ABFC84.

## Conflicts of Interest

The authors declare no conflicts of interest.

## Supporting information


**Video S1:** Pseudopodium in two cells of *Telonema blandense*. Video accelerated 230× times. Time elapsed = 2.73 h.

**Video S2:** Vacuole ejection in *Telonema blandense*. Video accelerated 230× times. Time elapsed = 1.37 h.

**Video S3:** Elongating structure in *Telonema subtile*. Video accelerated 10× times. Time elapsed = 90 s.

**Table S1:** All ribosomal sequence information and origin.

## Data Availability

The 18S rRNA gene sequences of BEAP0010, BEAP0082, BEAP0314, and BEAP0326 have been deposited in GenBank with the accession codes PV654196, PV654197, PV654198, and PV654195, respectively. Phylogenetic trees (and the data necessary to reproduce them), videos, and SEM images have been deposited in FigShare at DOI 10.6084/m9.figshare.29066438. Cell cultures containing BEAP0082, BEAP0314, and BEAP0326 (and mixed prey eukaryotes and bacteria) have been deposited in the Culture Collection of Algae and Protozoa (CCAP) with accession numbers CCAP 2621/1 (BEAP0326), CCAP 2134/1 (BEAP0314), and CCAP 2134/2 (BEAP0082). SEM stubs for BEAP0082, BEAP0314, and BEAP0326 have been deposited in the Marine Biological Reference Collections (CBMR) at the Institut de Ciències del Mar (ICM‐CSIC, Barcelona, Spain) under the catalog/accession numbers ICMCBMR000700, ICMCBMR000698, and ICMCBMR000699, respectively.
